# Supplementary material for: Passive acoustic methods for tracking the 3D movements of small cetaceans around marine structures
Source: PLoS One. 2020 May 29;15(5):e0229058. doi: 10.1371/journal.pone.0229058 (PMC7259614; doi:10.1371/journal.pone.0229058)
Supplement: Attachment — Submitted filename: Response to Reviewers.docx [file pone.0229058.s001.docx]

St Andrews

17 March, 2010

PONE-D-20-02520
Passive acoustic methods for tracking the 3D movements of small cetaceans around marine structures
PLOS ONE

Dear Dr Halliday,

Thank you for your responses and the reviewer responses to our manuscript, all of which have been most helpful in producing a revised and better piece of work. We hope that we have addressed these comments to your satisfaction and have uploaded correctly named documents and figures, including with track changes as well as this rebuttal letter. Our responses to specific comments are detailed below.

Yours Sincerely,

Douglas Gillespie

**Editor and reviewer comments in bold**, responses in plain text. Line numbers refer to lines in Revised Manuscript.docx

**Comments by editor

To enhance the reproducibility of your results, we recommend that if applicable you deposit your laboratory protocols in protocols.io, where a protocol can be assigned its own identifier (DOI) such that it can be cited independently in the future.**

Response: We don’t see that there is additional detail required for this paper which could go to protocols.io. However, we are considering a submission on a paper we’re writing on porpoise movement using the methods described in this paper.

**Journal Requirements:**

**When submitting your revision, we need you to address these additional requirements.**

**1. Please ensure that your manuscript meets PLOS ONE's style requirements, including those for file naming.**

Response: We have updated format of the author list / title page and adhered to the file naming requirements.

**2. In your Methods section, please provide additional location information of the study area, including geographic coordinates for the data set if available.**

Response: We have added the geographic coordinates (58°39'N 3°08'W) at line 241

**3. We note that you have stated that you will provide repository information for your data at acceptance. Should your manuscript be accepted for publication, we will hold it until you provide the relevant accession numbers or DOIs necessary to access your data. If you wish to make changes to your Data Availability statement, please describe these changes in your cover letter and we will update your Data Availability statement to reflect the information you provide.**

Response: Data have been uploaded to <https://doi.org/10.17630/de341ca6-6754-43ed-b1ac-a55aae6ccfaa>

**4. Your ethics statement must appear in the Methods section of your manuscript. If your ethics statement is written in any section besides the Methods, please move it to the Methods section and delete it from any other section. Please also ensure that your ethics statement is included in your manuscript, as the ethics section of your online submission will not be published alongside your manuscript.**

Response: The ethics statement has been moved to the start of the Methods section (lines 78-79) and removed from the acknowledgements.

**Additional Editor Comments (if provided):

Both reviewers and I agree that this paper is well written, and with a small number of relatively minor revisions, can be acceptable for publication. Please address all comments from both reviewers when revising your manuscript. I will note that Reviewer 2 asks why you didn't use whistles for localization. Given that you describe using the PAMGuard whistle detector, this is a relevant query. Why describe using the whistle detector if you aren't using the whistle data here? Additionally, I noted three small errors:**

Response: I agree that this is a valid query and I have added some text to the discussion to say that very few whistles were detected and that we’ve not attempted to localise them and indeed that accurate localisation may not even be possible for these sounds (lines 396 – 404).

**Line 39: the Smith 2000 reference is outdated when discussing increased anthropogenic structures in the marine environment over the past several decades, since that paper is already 20 years old.**

Response: (Line 40) I have replaced this with a reference to Stojanovic TA, Farmer CJQ. The development of world oceans & coasts and concepts of sustainability. Mar Policy. 2013;42: 157–165. doi:10.1016/j.marpol.2013.02.005

**Line 242: strange text, likely from your reference management software: "(Error! Reference source not found.)" - this is also noted by Reviewer 1.**

Response: I apologise for not adequately checking the generated pdf. It was fine in the submitted word doc. Is now fixed

**Line 267: "an Fourier transform" should be "a fast Fourier transform".**

Response. Agree. Have changed text (though interestingly, there are now Non FFT algorithms available that are almost as fast as the FFT, so there is really little need these days to restrict ourselves to FFT lengths that are a power of 2. Indeed, the Matlab FFT function will do a FT of any length, so this is something to watch for in future publications). Lines 277 - 278

**3. Have the authors made all data underlying the findings in their manuscript fully available?**

**The** [**PLOS Data policy**](http://www.plosone.org/static/policies.action#sharing) **requires authors to make all data underlying the findings described in their manuscript fully available without restriction, with rare exception (please refer to the Data Availability Statement in the manuscript PDF file). The data should be provided as part of the manuscript or its supporting information, or deposited to a public repository. For example, in addition to summary statistics, the data points behind means, medians and variance measures should be available. If there are restrictions on publicly sharing data—e.g. participant privacy or use of data from a third party—those must be specified.

Reviewer #1: Yes

Reviewer #2: No**

 Response: As stated earlier, data have been uploaded to DOI at :

**5. Review Comments to the Author

Reviewer #1:**

**Comment:
Maybe, in the conclusions the authors should indicate more explicitly how the information about fine-scale movements of cetaceans in the proximity of the turbines can be practically exploited for preventing cetaceans from possible collisions and consequent injuries.**

Response: I have added text to the discussion discussing the possibility of stopping turbines when animals are detected. Our initial priority is to assess whether there is a problem that needs solving rather than to change either turbine or animal behaviour. Implementing a turbine shut-down on detection system would be extremely challenging. (lines 427 to 434).

Major issues

**The level of automation and the skill of real-time detection of the system cannot be easily evinced by the text from the beginning. At line 116 the authors claim that "real-time processing" is conducted on shore using PAMguard software, but at line 124 it is said that the click detector was configured in such a way that the false positive rate is quite high and a post hoc manual screening was necessary to extract cetacean clicks.
From the description reported in Section 3 (“Offline Analysis”) it is clear that a big effort has been made by the authors in tuning the detection/tracking algorithms and in measuring the localization accuracy, but it's not clear if this has been subsequently implemented in the algorithms to make the system autonomous.
Only at the end of the paper ("Conclusions" section, line 395-405) it is asserted that at this moment the system is not totally automated and the developing of an automated encounter detection process is postponed to future studies**.

Response: These are good points, though in our defence, there are only 8 lines separating the two statements at lines 116 and 124. The real-time processing was used to reduce data volumes by a factor of about 300, then remaining data validated offline. These points are now addressed by a sentence added to the start of the Methods section (lines 75-77): “The system is semi-automatic, with real time detectors reducing amounts of stored data by several orders of magnitude, but operator screening of remaining sounds being required to select and confirm detections from the stored data”

**Since the level of automation (i.e. the ability to first recognize true positive source signals and then localize the source) and the response time (real-time aspects) of a PAM system are crucial points for marine mammals conservation purposes, the authors should clarify this point earlier in the text in order to avoid confusion in the reader.**

**Moreover, the authors should better emphasize how the information about fine-scale movements of cetaceans in the proximity of the turbines can be exploited for preventing cetaceans from possible collisions with moving turbine parts and consequent injuries. For example indicating if this method can be used for implementing a real-time monitoring system equipped with an alarm generation that could break the turbine blades in the presence of one or more individuals.**

Response: (We assume the reviewer means “brake” and not “break”). These two points are related and not very relevant to the paper. While I agree that the system as stands is not suitable for real time mitigation, a more fundamental problem than making a detection decision in real time would be the stopping of the turbine. The purpose of our current study is to establish whether or not there is a problem that needs solving, not so solve it. Talking informally with engineers, a braking system that could stop a turbine within a matter of seconds in response to a detection would be a major challenge. I comment on this further in the discussion (lines 417 to 434) and make it clear that the purpose of this system is for study of animals, not for mitigation (indeed, mitigation was deliberately not mentioned once in the original manuscript).


**Minor issues

- the authors should decide the format of the bibliography citations and use it all over the paper: sometimes the square bracket format (for ex [1]) and sometimes the (first author, year) format is used.**

Response: Apologies. This has been fixed.

**- line 61-63
considering that during operation time the turbine produces a high level of acoustic noise centered in a frequency band that partially overlaps with the one of the dolphin whistle (see fig.2), could the system detect dolphin whistle at acceptable percentage of success?**

Response: We don’t know. This is discussed at line 396 - 404.

**- line 64
been --> be**

Response: Corrected. (line 62)

**- line 90
... the sound can (be) localised**

Response: Corrected.

**- line 142-146:
<> The explanation of the division of clicks received at different clusters into 'possible groups' is slightly confusing. It can be evinced that the group assignment criterium is based on time constraint coming from the maximum time delay of a received click at different clusters, nominally max_Tdelay = cluster spacing/ sound speed in water. The authors should better clarify this part to avoid confusion by rephrasing the sentence and/or by supporting the sentence with a simple scheme (like the one in Fig.3)**

Response: We don’t think that a diagram will be useful here (there is one in Macaulay 2018) but based on this comment we have amended the text which is now a lot clearer (lines 150 - 160).

**- line 199-208
<>
Again a simple diagram reporting labeled Cartesian axis and cardinal points could better clarify the sentence.**

Response: We have added a diagram showing the coordinate system for the errors (new figure 2). Caption at lines 218 to 222

**- line 242
error on reference**

Response: Corrected.


**My major concern is that it mainly discussed the detection and underwater tracking by using echolocation clicks, and no data are presented for dolphin whistle detections. Additionally, the dolphin detection algorism used for porpoise echolocation detection is not clearly stated.**

Response: We have added text at lines 138 - 141 to give further information on the click detection and classification process.

**Some minor revision suggestions was following:
Line 26 localisation should be localization**

Response: We have used British English. We find nothing on the journal website requesting US English though are happy to change if requested by the editor.

**Line 52 reference (e.g. Johnson and Tyack, 2003) should following the format of the journal
Line 54, same problem as mentioned above
Line 63 reference should change the citing format
Line 65 same problem as mentioned above**

Response to above four points: Apologies. All corrected.

**Line 112 information of NI-9222, such as manufacture country and company should added**

Response: Have added National Instruments address. (Line 123)

**Line 133 Lewis et al., 2018 should modified to the journal required style
Line 138 same problem as mentioned above**
Response to above two points: Apologies. All corrected.

**While revising your submission, please upload your figure files to the Preflight Analysis and Conversion Engine (PACE) digital diagnostic tool,** [**https://pacev2.apexcovantage.com/**](https://pacev2.apexcovantage.com/)**. PACE helps ensure that figures meet PLOS requirements. To use PACE, you must first register as a user. Registration is free. Then, login and navigate to the UPLOAD tab, where you will find detailed instructions on how to use the tool. If you encounter any issues or have any questions when using PACE, please email us at** [**figures@plos.org**](mailto:figures@plos.org)**. Please note that Supporting Information files do not need this step.**

 All figures have been passed through PACE and uploaded in the correct format.
